# A Case of Asymptomatic Juvenile Emphysema

**DOI:** 10.31662/jmaj.2025-0042

**Published:** 2025-06-06

**Authors:** Taro Koba, Shuichi Matsuda, Miake Yamamoto, Mikio Takamori

**Affiliations:** 1 Department of Respiratory Medicine and Oncology, Tokyo Metropolitan Tama Medical Center, Tokyo, Japan

**Keywords:** premature birth, bronchopulmonary dysplasia, juvenile emphysema

A 15-year-old adolescent boy with a history of childhood asthma was referred to our hospital after emphysema was suspected on a routine chest radiograph. The patient was asymptomatic, and neither he nor his parents had a smoking history. The patient’s mother had childhood asthma. A chest radiograph showed increased lucency in the left upper lung (Figure 1), and computed tomography revealed scattered areas of panlobular emphysema (Figure 2). Alpha-1 antitrypsin was normal, and a pulmonary function test showed no significant obstructive impairment.

**Figure 1.**
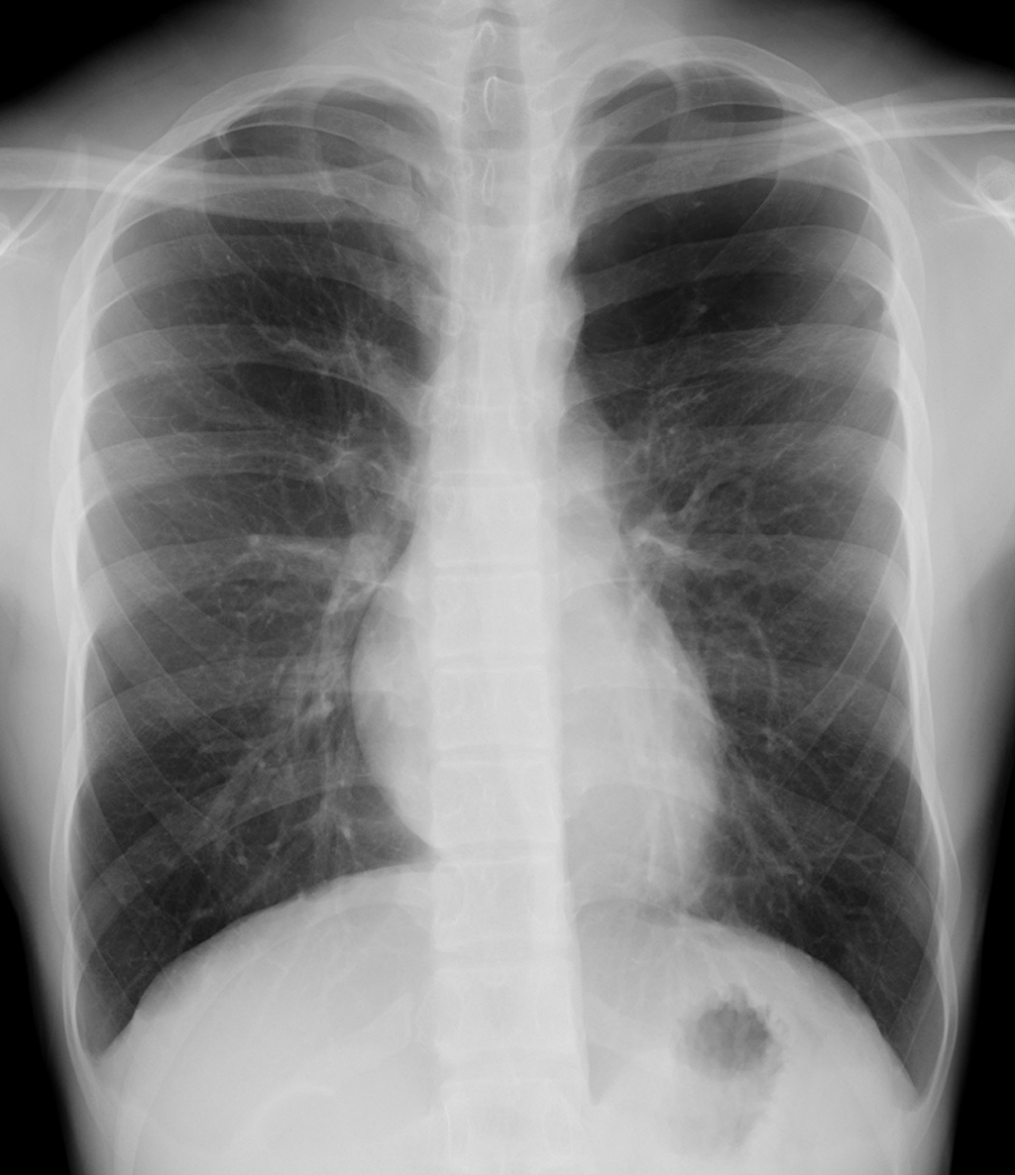
Chest radiograph showing increased radiolucency in the left upper lung field.

**Figure 2.**
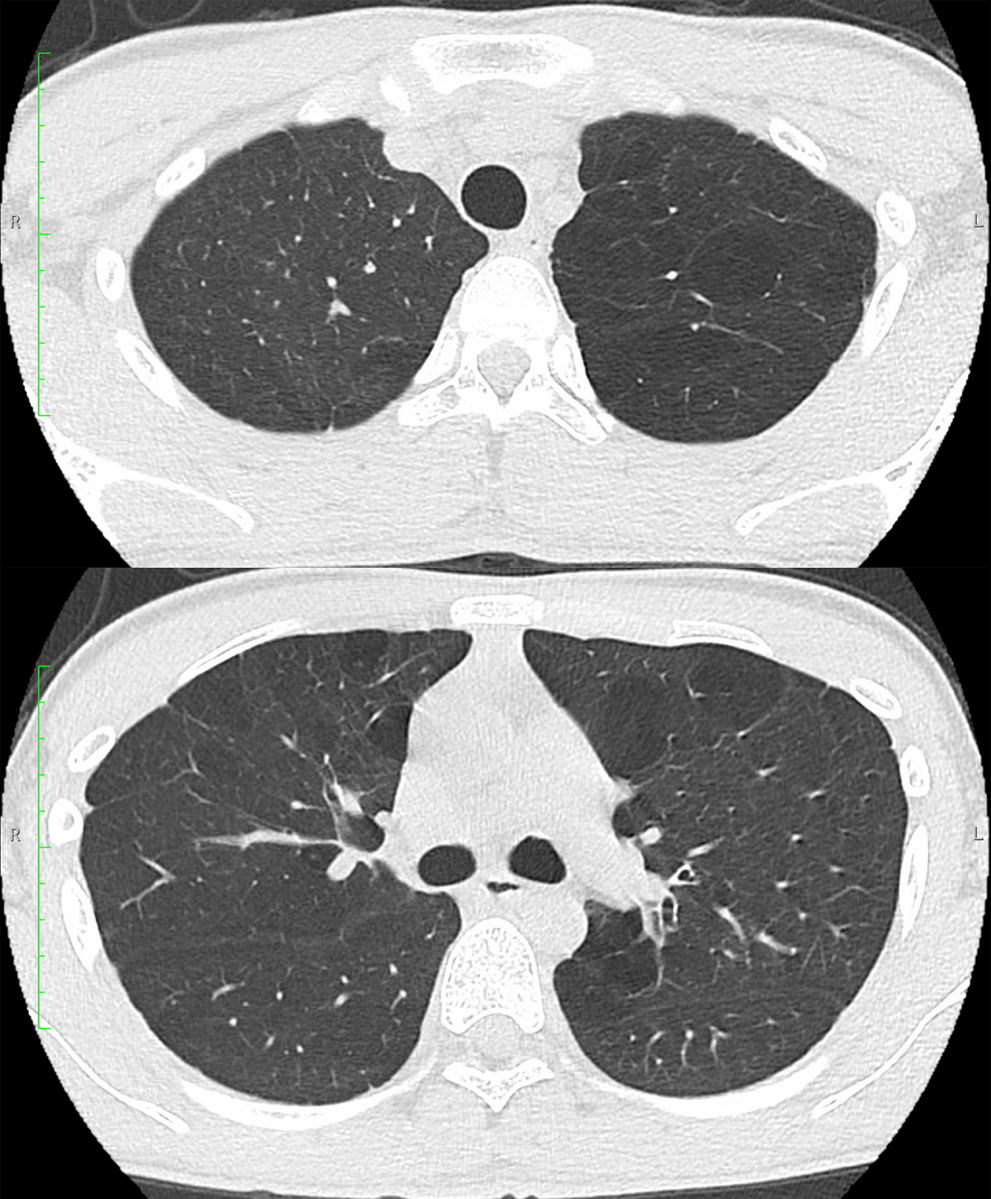
Chest computed tomography scan showing scattered panlobular emphysema predominantly in the left upper lobe.

During history-taking, it was revealed that the patient was born prematurely at 27 weeks. Infants who are extremely preterm are typically at high risk for respiratory failure; however, in this case, the patient required only a few days of noninvasive positive airway pressure. He did not require endotracheal surfactant therapy and had an uneventful respiratory course without bronchopulmonary dysplasia developing ^(1)^. A chest radiograph at the age of 1 year showed the same increased lucency (Figure 3), and emphysema linked to premature birth was diagnosed.

**Figure 3.**
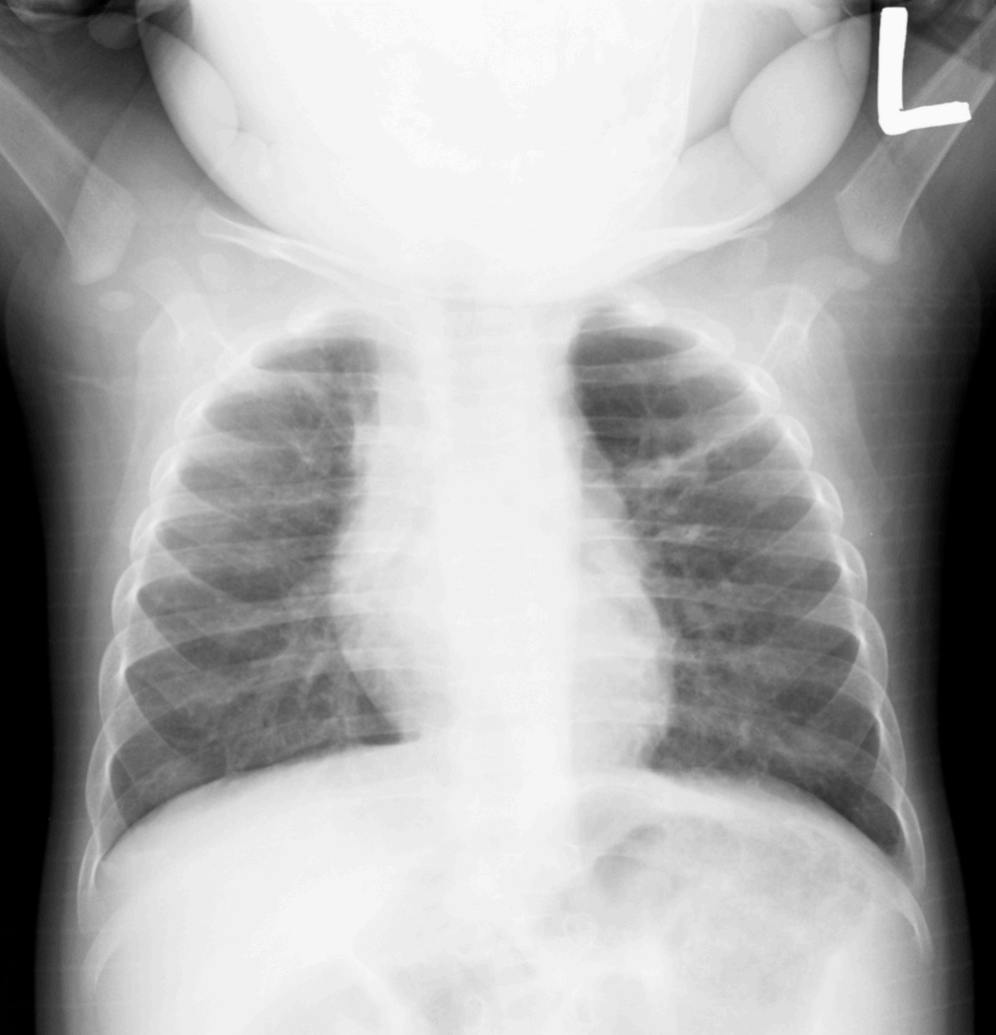
Chest radiograph at the age of 1 year showing similar increased radiolucency in the left upper lung field.

Premature birth is a risk factor for emphysema development ^(2)^. Thus, history-taking is necessary to determine whether a patient was born prematurely when assessing juvenile emphysema.

## Article Information

### Conflicts of Interest

None

### Author Contributions

All authors, TK, SM, MY, and MT, were involved in the diagnosis of this case, as well as in the writing of the paper, and all the authors meet the ICMJE recommended four criteria.

### Informed Consent

Informed consent was obtained from the patient for the publication of the details of his case.
